# Ten simple rules for good research practice

**DOI:** 10.1371/journal.pcbi.1010139

**Published:** 2022-06-23

**Authors:** Simon Schwab, Perrine Janiaud, Michael Dayan, Valentin Amrhein, Radoslaw Panczak, Patricia M. Palagi, Lars G. Hemkens, Meike Ramon, Nicolas Rothen, Stephen Senn, Eva Furrer, Leonhard Held

**Affiliations:** 1 Center for Reproducible Science, University of Zurich, Zurich, Switzerland; 2 Epidemiology, Biostatistics and Prevention Institute, University of Zurich, Zurich, Switzerland; 3 Department of Clinical Research, University Hospital Basel, University of Basel, Basel, Switzerland; 4 Human Neuroscience Platform, Fondation Campus Biotech Geneva, Geneva, Switzerland; 5 Department of Environmental Sciences, Zoology, University of Basel, Basel, Switzerland; 6 Institute of Social and Preventive Medicine, University of Bern, Bern, Switzerland; 7 SIB Training Group, SIB Swiss Institute of Bioinformatics, Lausanne, Switzerland; 8 Meta-Research Innovation Center at Stanford (METRICS), Stanford University, Stanford, California, United States of America; 9 Meta-Research Innovation Center Berlin (METRIC-B), Berlin Institute of Health, Berlin, Germany; 10 Applied Face Cognition Lab, University of Lausanne, Lausanne, Switzerland; 11 Faculty of Psychology, UniDistance Suisse, Brig, Switzerland; 12 Statistical Consultant, Edinburgh, United Kingdom

This is a *PLOS Computational Biology* Methods paper.

## Introduction

The lack of research reproducibility has caused growing concern across various scientific fields [1–5]. Today, there is widespread agreement, within and outside academia, that scientific research is suffering from a reproducibility crisis [6,7]. Researchers reach different conclusions—even when the same data have been processed—simply due to varied analytical procedures [8,9]. As we continue to recognize this problematic situation, some major causes of irreproducible research have been identified. This, in turn, provides the foundation for improvement by identifying and advocating for good research practices (GRPs). Indeed, powerful solutions are available, for example, preregistration of study protocols and statistical analysis plans, sharing of data and analysis code, and adherence to reporting guidelines. Although these and other best practices may facilitate reproducible research and increase trust in science, it remains the responsibility of researchers themselves to actively integrate them into their everyday research practices.

Contrary to ubiquitous specialized training, cross-disciplinary courses focusing on best practices to enhance the quality of research are lacking at universities and are urgently needed. The intersections between disciplines offer a space for peer evaluation, mutual learning, and sharing of best practices. In medical research, interdisciplinary work is inevitable. For example, conducting clinical trials requires experts with diverse backgrounds, including clinical medicine, pharmacology, biostatistics, evidence synthesis, nursing, and implementation science. Bringing researchers with diverse backgrounds and levels of experience together to exchange knowledge and learn about problems and solutions adds value and improves the quality of research.

The present selection of rules was based on our experiences with teaching GRP courses at the University of Zurich, our course participants’ feedback, and the views of a cross-disciplinary group of experts from within the Swiss Reproducibility Network (www.swissrn.org). The list is neither exhaustive, nor does it aim to address and systematically summarize the wide spectrum of issues including research ethics and legal aspects (e.g., related to misconduct, conflicts of interests, and scientific integrity). Instead, we focused on practical advice at the different stages of everyday research: from planning and execution to reporting of research. For a more comprehensive overview on GRPs, we point to the United Kingdom’s Medical Research Council’s guidelines [10] and the Swedish Research Council’s report [11]. While the discussion of the rules may predominantly focus on clinical research, much applies, in principle, to basic biomedical research and research in other domains as well.

**Fig 1:**
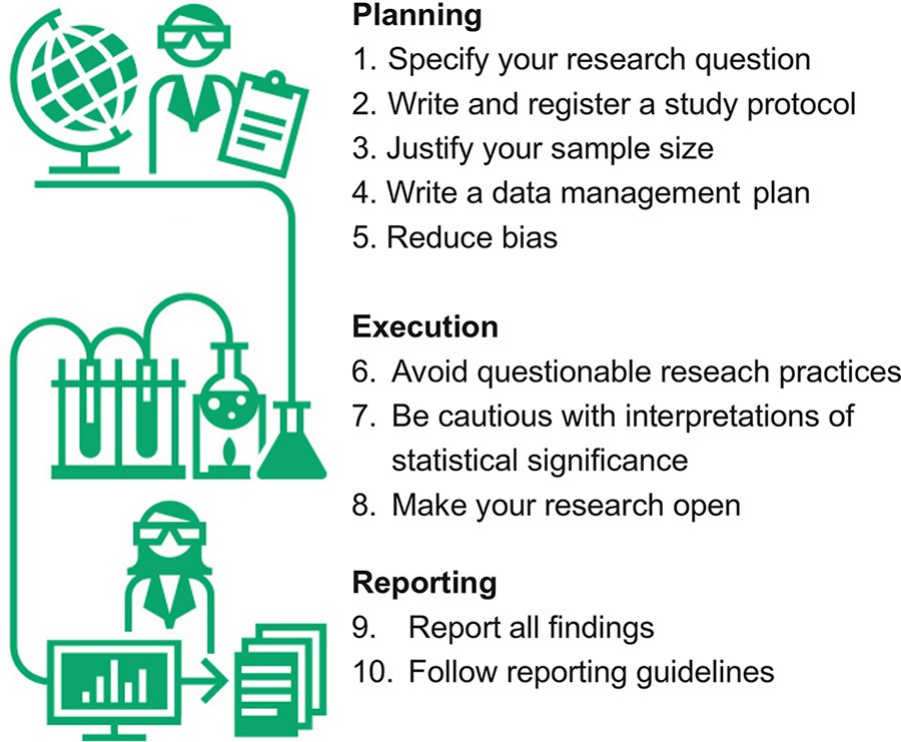
The 10 simple rules for GRP grouped into planning, execution, and reporting of research. GRP, good research practices.

The 10 proposed rules can serve multiple purposes: an introduction for researchers to relevant concepts to improve research quality, a primer for early-career researchers who participate in our GRP courses, or a starting point for lecturers who plan a GRP course at their own institutions. The 10 rules are grouped according to planning (5 rules), execution (3 rules), and reporting of research (2 rules); see Fig 1. These principles can (and should) be implemented as a habit in everyday research, just like toothbrushing.

## Research planning

### Rule 1: Specify your research question

Coming up with a research question is not always simple and may take time. A successful study requires a narrow and clear research question. In evidence-based research, prior studies are assessed in a systematic and transparent way to identify a research gap for a new study that answers a question that matters [12]. Papers that provide a comprehensive overview of the current state of research in the field are particularly helpful—for example, systematic reviews. Perspective papers may also be useful, for example, there is a paper with the title “SARS-CoV-2 and COVID-19: The most important research questions.” However, a systematic assessment of research gaps deserves more attention than opinion-based publications.

In the next step, a vague research question should be further developed and refined. In clinical research and evidence-based medicine, there is an approach called population, intervention, comparator, outcome, and time frame (PICOT) with a set of criteria that can help framing a research question [13]. From a well-developed research question, subsequent steps will follow, which may include the exact definition of the population, the outcome, the data to be collected, and the sample size that is required. It may be useful to find out if other researchers find the idea interesting as well and whether it might promise a valuable contribution to the field. However, actively involving the public or the patients can be a more effective way to determine what research questions matter.

The level of details in a research question also depends on whether the planned research is confirmatory or exploratory. In contrast to confirmatory research, exploratory research does not require a well-defined hypothesis from the start. Some examples of exploratory experiments are those based on omics and multi-omics experiments (genomics, bulk RNA-Seq, single-cell, etc.) in systems biology and connectomics and whole-brain analyses in brain imaging. Both exploration and confirmation are needed in science, and it is helpful to understand their strengths and limitations [14,15].

### Rule 2: Write and register a study protocol

In clinical research, registration of clinical trials has become a standard since the late 1990 and is now a legal requirement in many countries. Such studies require a study protocol to be registered, for example, with ClinicalTrials.gov, the European Clinical Trials Register, or the World Health Organization’s International Clinical Trials Registry Platform. Similar effort has been implemented for registration of systematic reviews (PROSPERO). Study registration has also been proposed for observational studies [16] and more recently in preclinical animal research [17] and is now being advocated across disciplines under the term “preregistration” [18,19].

Study protocols typically document at minimum the research question and hypothesis, a description of the population, the targeted sample size, the inclusion/exclusion criteria, the study design, the data collection, the data processing and transformation, and the planned statistical analyses. The registration of study protocols reduces publication bias and hindsight bias and can safeguard honest research and minimize waste of research [20–22]. Registration ensures that studies can be scrutinized by comparing the reported research with what was actually planned and written in the protocol, and any discrepancies may indicate serious problems (e.g., outcome switching).

Note that registration does not mean that researchers have no flexibility to adapt the plan as needed. Indeed, new or more appropriate procedures may become available or known only after registration of a study. Therefore, a more detailed statistical analysis plan can be amended to the protocol before the data are observed or unblinded [23,24]. Likewise, registration does not exclude the possibility to conduct exploratory data analyses; however, they must be clearly reported as such.

To go even further, registered reports are a novel article type that incentivize high-quality research—irrespective of the ultimate study outcome [25,26]. With registered reports, peer-reviewers decide before anyone knows the results of the study, and they have a more active role in being able to influence the design and analysis of the study. Journals from various disciplines increasingly support registered reports [27].

Naturally, preregistration and registered reports also have their limitations and may not be appropriate in a purely hypothesis-generating (explorative) framework. Reports of exploratory studies should indeed not be molded into a confirmatory framework; appropriate rigorous reporting alternatives have been suggested and start to become implemented [28,29].

### Rule 3: Justify your sample size

Early-career researchers in our GRP courses often identify sample size as an issue in their research. For example, they say that they work with a low number of samples due to slow growth of cells, or they have a limited number of patient tumor samples due to a rare disease. But if your sample size is too low, your study has a high risk of providing a false negative result (type II error). In other words, you are unlikely to find an effect even if there truly was an effect.

Unfortunately, there is more bad news with small studies. When an effect from a small study was selected for drawing conclusions because it was statistically significant, low power increases the probability that an effect size is overestimated [30,31]. The reason is that with low power, studies that due to sampling variation find larger (overestimated) effects are much more likely to be statistically significant than those that happen to find smaller (more realistic) effects [30,32,33]. Thus, in such situations, effect sizes are often overestimated. For the phenomenon that small studies often report more extreme results (in meta-analyses), the term “small-study effect” was introduced [34]. In any case, an underpowered study is a problematic study, no matter the outcome.

In conclusion, small sample sizes can undermine research, but when is a study too small? For one study, a total of 50 patients may be fine, but for another, 1,000 patients may be required. How large a study needs to be designed requires an appropriate sample size calculation. Appropriate sample size calculation ensures that enough data are collected to ensure sufficient statistical power (the probability to reject the null hypothesis when it is in fact false).

Low-powered studies can be avoided by performing a sample size calculation to find out the required sample size of the study. This requires specifying a primary outcome variable and the magnitude of effect you are interested in (among some other factors); in clinical research, this is often the minimal clinically relevant difference. The statistical power is often set at 80% or larger. A comprehensive list of packages for sample size calculation are available [35], among them the R package “pwr” [36]. There are also many online calculators available, for example, the University of Zurich’s “SampleSizeR” [37].

A worthwhile alternative for planning the sample size that puts less emphasis on null hypothesis testing is based on the desired precision of the study; for example, one can calculate the sample size that is necessary to obtain a desired width of a confidence interval for the targeted effect [38–40]. A general framework to sample size justification beyond a calculation-only approach has been proposed [41]. It is also worth mentioning that some study types have other requirements or need specific methods. In diagnostic testing, one would need to determine the anticipated minimal sensitivity or specificity; in prognostic research, the number of parameters that can be used to fit a prediction model given a fixed sample size should be specified. Designs can also be so complex that a simulation (Monte Carlo method) may be required.

Sample size calculations should be done under different assumptions, and the largest estimated sample size is often the safer bet than a best-case scenario. The calculated sample size should further be adjusted to allow for possible missing data. Due to the complexity of accurately calculating sample size, researchers should strongly consider consulting a statistician early in the study design process.

### Rule 4: Write a data management plan

In 2020, 2 Coronavirus Disease 2019 (COVID-19) papers in leading medical journals were retracted after major concerns about the data were raised [42]. Today, raw data are more often recognized as a key outcome of research along with the paper. Therefore, it is important to develop a strategy for the life cycle of data, including suitable infrastructure for long-term storage.

The data life cycle is described in a data management plan: a document that describes what data will be collected and how the data will be organized, stored, handled, and protected during and after the end of the research project. Several funders require a data management plan in grant submissions, and publishers like PLOS encourage authors to do so as well. The Wellcome Trust provides guidance in the development of a data management plan, including real examples from neuroimaging, genomics, and social sciences [43]. However, projects do not always allocate funding and resources to the actual implementation of the data management plan.

The Findable, Accessible, Interoperable, and Reusable (FAIR) data principles promote maximal use of data and enable machines to access and reuse data with minimal human intervention [44]. FAIR principles require the data to be retained, preserved, and shared preferably with an immutable unique identifier and a clear usage license. Appropriate metadata will help other researchers (or machines) to discover, process, and understand the data. However, requesting researchers to fully comply with the FAIR data principles in every detail is an ambitious goal.

Multidisciplinary data repositories that support FAIR are, for example, Dryad (datadryad.org https://datadryad.org/), EUDAT (www.eudat.eu), OSF (osf.io https://osf.io/), and Zenodo (zenodo.org https://zenodo.org/). A number of institutional and field-specific repositories may also be suitable. However, sometimes, authors may not be able to make their data publicly available for legal or ethical reasons. In such cases, a data user agreement can indicate the conditions required to access the data. Journals highlight what are acceptable and what are unacceptable data access restrictions and often require a data availability statement.

Organizing the study artifacts in a structured way greatly facilitates the reuse of data and code within and outside the lab, enhancing collaborations and maximizing the research investment. Support and courses for data management plans are sometimes available at universities. Another 10 simple rules paper for creating a good data management plan is dedicated to this topic [45].

### Rule 5: Reduce bias

Bias is a distorted view in favor of or against a particular idea. In statistics, bias is a systematic deviation of a statistical estimate from the (true) quantity it estimates. Bias can invalidate our conclusions, and the more bias there is, the less valid they are. For example, in clinical studies, bias may mislead us into reaching a causal conclusion that the difference in the outcomes was due to the intervention or the exposure. This is a big concern, and, therefore, the risk of bias is assessed in clinical trials [46] as well as in observational studies [47,48].

There are many different forms of bias that can occur in a study, and they may overlap (e.g., allocation bias and confounding bias) [49]. Bias can occur at different stages, for example, immortal time bias in the design of the study, information bias in the execution of the study, and publication bias in the reporting of research. Understanding bias allows us researchers to remain vigilant of potential sources of bias when peer-reviewing and designing own studies. We summarized some common types of bias and some preventive steps in Table 1, but many other forms of bias exist; for a comprehensive overview, see the Oxford University’s Catalogue of Bias [50].

**Table 1:** Common types of bias that can affect a research study and some measures that may prevent them.

Name	Explanation	Prevention
Allocation bias	Systematic difference in the assignment of participants to the treatment and control group in a clinical trial. For example, the investigator knows or can predict which intervention the next eligible patient is supposed to receive due to poorly concealed randomization.	- Randomization with allocation concealment
Attrition bias	Attrition occurs when participants leave during a study that aims to explore the effect of continuous exposure (dropouts or withdrawal). For example, more dropouts of patients randomized to an aggressive cancer treatment.	- Good investigator–patient communication - Accessibility of clinics - Incentives to continue
Confounding bias	An artificial association between an exposure and an outcome because another variable is related to both the exposure and outcome. For example, lung cancer risk in coffee drinkers is evaluated, ignoring smoking status (smoking is associated with both coffee drinking and cancer). A challenge is that many confounders are unknown and/or not measured.	- Randomization (can address unmeasured confounders) When randomization is not possible: - Restriction to one level of the confounder - Matching on the levels of the confounder - Stratification and analysis within strata - Propensity score matching
Immortal time bias	Survival beyond a certain time point is necessary in order to be exposed (participants are “immortal” in that time period). For example, discharged patients are analyzed but were included in the treatment group only if they filled a prescription for a drug 90 days after discharge from hospital.	- Group assignment at time zero - Time-dependent analysis may be used
Information bias	Bias that arises from systematic differences in the collection, recall, recording, or handling of information. For example, blood pressure in the treatment arm is measured in the morning and for the control arm in the evening.	- Standardized data collection - Data collection independent from exposure or outcome (e.g., by blinding of intervention status/exposure) - Use of objective measurements
Publication bias	Occurs when only studies with a positive or negative result are published. Affects meta-analyses from systematic reviews and harms evidence-based medicine	- Writing a study protocol and preregistration - Publishing study protocol or registered report - Following reporting guidelines

For a comprehensive collection, see catalogofbias.org.

Here are some noteworthy examples of study bias from the literature: An example of information bias was observed when in 1998 an alleged association between the measles, mumps, and rubella (MMR) vaccine and autism was reported. Recall bias (a subtype of information bias) emerged when parents of autistic children recalled the onset of autism after an MMR vaccination more often than parents of similar children who were diagnosed prior to the media coverage of that controversial and meanwhile retracted study [51]. A study from 2001 showed better survival for academy award-winning actors, but this was due to immortal time bias that favors the treatment or exposure group [52,53]. A study systematically investigated self-reports about musculoskeletal symptoms and found the presence of information bias. The reason was that participants with little computer-time overestimated, and participants with a lot of computer-time spent underestimated their computer usage [54].

Information bias can be mitigated by using objective rather than subjective measurements. Standardized operating procedures (SOP) and electronic lab notebooks additionally help to follow well-designed protocols for data collection and handling [55]. Despite the failure to mitigate bias in studies, complete descriptions of data and methods can at least allow the assessment of risk of bias.

## Research execution

### Rule 6: Avoid questionable research practices

Questionable research practices (QRPs) can lead to exaggerated findings and false conclusions and thus lead to irreproducible research. Often, QRPs are used with no bad intentions. This becomes evident when methods sections explicitly describe such procedures, for example, to increase the number of samples until statistical significance is reached that supports the hypothesis. Therefore, it is important that researchers know about QRPs in order to recognize and avoid them.

Several questionable QRPs have been named [56,57]. Among them are low statistical power, pseudoreplication, repeated inspection of data, *p*-hacking [58], selective reporting, and hypothesizing after the results are known (HARKing).

The first 2 QRPs, low statistical power and pseudoreplication, can be prevented by proper planning and designing of studies, including sample size calculation and appropriate statistical methodology to avoid treating data as independent when in fact they are not. Statistical power is not equal to reproducibility, but statistical power is a precondition of reproducibility as the lack thereof can result in false negative as well as false positive findings (see Rule 3).

In fact, a lot of QRP can be avoided with a study protocol and statistical analysis plan. Preregistration, as described in Rule 2, is considered best practice for this purpose. However, many of these issues can additionally be rooted in institutional incentives and rewards. Both funding and promotion are often tied to the quantity rather than the quality of the research output. At universities, still only few or no rewards are given for writing and registering protocols, sharing data, publishing negative findings, and conducting replication studies. Thus, a wider “culture change” is needed.

### Rule 7: Be cautious with interpretations of statistical significance

It would help if more researchers were familiar with correct interpretations and possible misinterpretations of statistical tests, *p*-values, confidence intervals, and statistical power [59,60]. A statistically significant *p*-value does not necessarily mean that there is a clinically or biologically relevant effect. Specifically, the traditional dichotomization into statistically significant (*p* < 0.05) versus statistically nonsignificant (*p* ≥ 0.05) results is seldom appropriate, can lead to cherry-picking of results and may eventually corrupt science [61]. We instead recommend reporting exact *p*-values and interpreting them in a graded way in terms of the compatibility of the null hypothesis with the data [62,63]. Moreover, a *p*-value around 0.05 (e.g., 0.047 or 0.055) provides only little information, as is best illustrated by the associated replication power: The probability that a hypothetical replication study of the same design will lead to a statistically significant result is only 50% [64] and is even lower in the presence of publication bias and regression to the mean (the phenomenon that effect estimates in replication studies are often smaller than the estimates in the original study) [65]. Claims of novel discoveries should therefore be based on a smaller *p*-value threshold (e.g., *p* < 0.005) [66], but this really depends on the discipline (genome-wide screenings or studies in particle physics often apply much lower thresholds).

Generally, there is often too much emphasis on *p*-values. A statistical index such as the *p*-value is just the final product of an analysis, the tip of the iceberg [67]. Statistical analyses often include many complex stages, from data processing, cleaning, transformation, addressing missing data, modeling, to statistical inference. Errors and pitfalls can creep in at any stage, and even a tiny error can have a big impact on the result [68]. Also, when many hypothesis tests are conducted (multiple testing), false positive rates may need to be controlled to protect against wrong conclusions, although adjustments for multiple testing are debated [69–71].

Thus, a *p*-value alone is not a measure of how credible a scientific finding is [72]. Instead, the quality of the research must be considered, including the study design, the quality of the measurement, and the validity of the assumptions that underlie the data analysis [60,73]. Frameworks exist that help to systematically and transparently assess the certainty in evidence; the most established and widely used one is Grading of Recommendations, Assessment, Development and Evaluations (GRADE; www.gradeworkinggroup.org) [74].

Training in basic statistics, statistical programming, and reproducible analyses and better involvement of data professionals in academia is necessary. University departments sometimes have statisticians that can support researchers. Importantly, statisticians need to be involved early in the process and on an equal footing and not just at the end of a project to perform the final data analysis.

### Rule 8: Make your research open

In reality, science often lacks transparency. Open science makes the process of producing evidence and claims transparent and accessible to others [75]. Several universities and research funders have already implemented open science roadmaps to advocate free and public science as well as open access to scientific knowledge, with the aim of further developing the credibility of research. Open research allows more eyes to see it and critique it, a principle similar to the “Linus’s law” in software development, which says that if there are enough people to test a software, most bugs will be discovered.

As science often progresses incrementally, writing and sharing a study protocol and making data and methods readily available is crucial to facilitate knowledge building. The Open Science Framework (osf.io) is a free and open-source project management tool that supports researchers throughout the entire project life cycle. OSF enables preregistration of study protocols and sharing of documents, data, analysis code, supplementary materials, and preprints.

To facilitate reproducibility, a research paper can link to data and analysis code deposited on OSF. Computational notebooks are now readily available that unite data processing, data transformations, statistical analyses, figures and tables in a single document (e.g., R Markdown, Jupyter); see also the 10 simple rules for reproducible computational research [76]. Making both data and code open thus minimizes waste of funding resources and accelerates science.

Open science can also advance researchers’ careers, especially for early-career researchers. The increased visibility, retrievability, and citations of datasets can all help with career building [77]. Therefore, institutions should provide necessary training, and hiring committees and journals should align their core values with open science, to attract researchers who aim for transparent and credible research [78].

## Research reporting

### Rule 9: Report all findings

Publication bias occurs when the outcome of a study influences the decision whether to publish it. Researchers, reviewers, and publishers often find nonsignificant study results not interesting or worth publishing. As a consequence, outcomes and analyses are only selectively reported in the literature [79], also known as the file drawer effect [80].

The extent of publication bias in the literature is illustrated by the overwhelming frequency of statistically significant findings [81]. A study extracted *p*-values from MEDLINE and PubMed Central and showed that 96% of the records reported at least 1 statistically significant *p*-value [82], which seems implausible in the real world. Another study plotted the distribution of more than 1 million *z*-values from Medline, revealing a huge gap from −2 to 2 [83]. Positive studies (i.e., statistically significant, perceived as striking or showing a beneficial effect) were 4 times more likely to get published than negative studies [84].

Often a statistically nonsignificant result is interpreted as a “null” finding. But a nonsignificant finding does not necessarily mean a null effect; absence of evidence is not evidence of absence [85]. An individual study may be underpowered, resulting in a nonsignificant finding, but the cumulative evidence from multiple studies may indeed provide sufficient evidence in a meta-analysis. Another argument is that a confidence interval that contains the null value often also contains non-null values that may be of high practical importance. Only if all the values inside the interval are deemed unimportant from a practical perspective, then it may be fair to describe a result as a null finding [61]. We should thus never report “no difference” or “no association” just because a *p*-value is larger than 0.05 or, equivalently, because a confidence interval includes the “null” [61].

On the other hand, studies sometimes report statistically nonsignificant results with “spin” to claim that the experimental treatment is beneficial, often by focusing their conclusions on statistically significant differences on secondary outcomes despite a statistically nonsignificant difference for the primary outcome [86,87].

Findings that are not being published have a tremendous impact on the research ecosystem, distorting our knowledge of the scientific landscape by perpetuating misconceptions, and jeopardizing judgment of researchers and the public trust in science. In clinical research, publication bias can mislead care decisions and harm patients, for example, when treatments appear useful despite only minimal or even absent benefits reported in studies that were not published and thus are unknown to physicians [88]. Moreover, publication bias also directly affects the formulation and proliferation of scientific theories, which are taught to students and early-career researchers, thereby perpetuating biased research from the core. It has been shown in modeling studies that unless a sufficient proportion of negative studies are published, a false claim can become an accepted fact [89] and the false positive rates influence trustworthiness in a given field [90].

In sum, negative findings are undervalued. They need to be more consistently reported at the study level or be systematically investigated at the systematic review level. Researchers have their share of responsibilities, but there is clearly a lack of incentives from promotion and tenure committees, journals, and funders.

### Rule 10: Follow reporting guidelines

Study reports need to faithfully describe the aim of the study and what was done, including potential deviations from the original protocol, as well as what was found. Yet, there is ample evidence of discrepancies between protocols and research reports, and of insufficient quality of reporting [79,91–95]. Reporting deficiencies threaten our ability to clearly communicate findings, replicate studies, make informed decisions, and build on existing evidence, wasting time and resources invested in the research [96].

Reporting guidelines aim to provide the minimum information needed on key design features and analysis decisions, ensuring that findings can be adequately used and studies replicated. In 2008, the Enhancing the QUAlity and Transparency Of Health Research (EQUATOR) network was initiated to provide reporting guidelines for a variety of study designs along with guidelines for education and training on how to enhance quality and transparency of health research. Currently, there are 468 reporting guidelines listed in the network; see the most prominent guidelines in Table 2. Furthermore, following the ICMJE recommendations, medical journals are increasingly endorsing reporting guidelines [97], in some cases making it mandatory to submit the appropriate reporting checklist along with the manuscript.

**Table 2:** Examples of reporting guidelines for different study types.

Guideline name	Study type
ARRIVE	Animal experiments
CONSORT	Randomized trials
STROBE	Observational studies
PRISMA	Systematic reviews
SPIRIT	Study protocols
STARD/TRIPOID	Diagnostic/prognostic studies

The EQUATOR Network is a library with more than 400 reporting guidelines in health research (www.equator-network.org).

The use of reporting guidelines and journal endorsement has led to a positive impact on the quality and transparency of research reporting, but improvement is still needed to maximize the value of research [98,99].

## Conclusions

Originally, this paper targeted early-career researchers; however, throughout the development of the rules, it became clear that the present recommendations can serve all researchers irrespective of their seniority. We focused on practical guidelines for planning, conducting, and reporting of research. Others have aligned GRP with similar topics [100,101]. Even though we provide 10 simple rules, the word “simple” should not be taken lightly. Putting the rules into practice usually requires effort and time, especially at the beginning of a research project. However, time can also be redeemed, for example, when certain choices can be justified to reviewers by providing a study protocol or when data can be quickly reanalyzed by using computational notebooks and dynamic reports.

Researchers have field-specific research skills, but sometimes are not aware of best practices in other fields that can be useful. Universities should offer cross-disciplinary GRP courses across faculties to train the next generation of scientists. Such courses are an important building block to improve the reproducibility of science.
